# A systematic review of the effects of brain-computer interface on lower limb motor function, balance function, and activities of daily living in stroke patients

**DOI:** 10.3389/fnins.2025.1641843

**Published:** 2026-01-12

**Authors:** Xiaozhen Guo, Pan Li, Hairong Liu, Song Ding

**Affiliations:** 1 Department of Physical Education, Tongji University, Shanghai, China; 2 School of Athletic Performance, Shanghai University of Sport, Shanghai, China; 3 Department of Physical Education, Shanghai International Studies University, Shanghai, China; 4 Department of Physical Education, Shanghai Jiao Tong University, Shanghai, China

**Keywords:** brain-computer interface, stroke, lower limb motor function, balance function, activities of daily living, systematic review

## Abstract

**Objective:**

To systematically evaluate the effects of brain-computer interface (BCI) technology on lower limb motor function, balance function, and activities of daily living in stroke patients.

**Methods:**

This study followed the PRISMA guidelines and searched PubMed, Web of Science, EMbase, The Cochrane Library, CNKI, Wanfang, and VIP databases, with an additional manual search. The search period was from database inception to March 2024. The PEDro scale was used to assess the quality of the studies, the GRADE system was applied to evaluate the evidence quality for outcome measures, and Meta-analysis was conducted using Stata 17.0 software.

**Results:**

The systematic review included nine studies. The methodological quality, assessed using the PEDro scale, yielded an average score of 6.9, which corresponds to a moderate-to-low certainty of evidence. The Meta-analysis showed that BCI technology significantly improved lower limb motor function (MD = 3.52, 95% CI [2.03, 5.00], *p* < 0.001) and activities of daily living (MD = 6.08, 95% CI [1.81, 10.35], *p* = 0.01), but had no significant effect on balance function (MD = 4.82, 95% CI [−1.53, 11.16], *p* = 0.14). Subgroup analysis showed that the effect size in the acute and subacute phases was 3.89, and in the recovery phase, it was 3.12, both of which were statistically significant. In terms of intervention methods, the effect size for MI-BCI was 2.73, and for BCI-Robot, it was 4.60, both statistically significant. Regarding intervention dosage, the effect size for 2.5–10 h was 2.60, and for 12–20 h, it was 5.46, both statistically significant.

**Conclusion:**

Current evidence suggests that BCI-based interventions have a beneficial effect on lower limb motor function and activities of daily living in stroke patients. Interventions initiated during the acute or subacute phase, with a total dose exceeding 12 h, appear to be associated with superior outcomes. However, the certainty of this evidence is moderate to low, necessitating further validation. Future research should prioritize large-scale, high-quality randomized controlled trials to definitively establish the efficacy of BCI technology and elucidate its optimal implementation protocols.

## 1 Introduction

Stroke is a common acute neurological disorder caused by cerebrovascular lesions, characterized by high incidence, high disability rate, and low cure rate (Fang et al., 2018). Approximately 6.55 million people die from stroke each year globally, and over 100 million people live with lifelong disabilities due to stroke. It is one of the leading causes of death and disability worldwide, with an increasing prevalence trend (GBD 2019 Stroke Collaborators, 2021). Complete recovery of neurological deficits after stroke is extremely difficult. Although current treatments can effectively salvage the ischemic penumbra and reduce mortality in the acute phase, the ability to repair established neural damage remains very limited (Marques et al., 2019). As a result, most stroke patients experience varying degrees of limb dysfunction, with lower limb motor dysfunction being a common sequela, severely impacting daily living abilities and quality of life (Wang et al., 2022). Although traditional rehabilitation therapies can help restore motor function to some extent, they still have numerous limitations, such as long treatment durations, slow effects, and low patient engagement (Smithard, 2003). Therefore, exploring new rehabilitation technologies, especially those that can directly affect the brain and promote neural plasticity, is of great significance for improving lower limb motor function recovery after stroke.

Brain-Computer Interface (BCI) decodes brain neural signals (e.g., movement-related cortical potentials) directly, converting them into control commands to bypass damaged neural pathways and drive external devices or stimulation systems, thereby helping patients with motor dysfunction rebuild communication and control abilities (Wolpaw et al., 2002). Research shows that BCI can specifically activate movement-related brain areas, promoting neural function remodeling and recovery (Qiu et al., 2023). For example, the associative BCI developed by Mrachacz-Kersting et al. (2016), by precisely matching the timing of movement intention and peripheral electrical stimulation, successfully induced increased excitability of the corticospinal tract and improved lower limb motor function in chronic stroke patients, providing mechanistic evidence for BCI-induced neural plasticity. In rehabilitation applications, BCI-controlled functional electrical stimulation can effectively activate the motor cortex and promote lower limb function recovery (Chung et al., 2015). Additionally, BCI training has been shown to improve patients’ activities of daily living and cognitive function, enhancing overall quality of life (Cheng et al., 2020). The real-time neurofeedback mechanism based on Hebbian theory further enhances the specificity of the training and patient engagement, thereby improving rehabilitation outcomes (Belkacem et al., 2020). These characteristics make BCI a mechanistically clear and promising new approach for post-stroke lower limb rehabilitation (Bockbrader et al., 2018).

BCI has shown potential in the rehabilitation of lower limb motor function in stroke patients, but its clinical application is still in the early stages, and long-term efficacy and optimal intervention parameters have yet to be determined. Therefore, this study systematically evaluates the rehabilitation effects of BCI on lower limb motor function, balance function, and activities of daily living in stroke patients through Meta-analysis, and explores key factors influencing its efficacy, aiming to provide empirical evidence for the clinical translation and optimization of this technology.

## 2 Research methods

### 2.1 Research protocol and registration

This study was reported according to the PRISMA guidelines (Preferred Reporting Items for Systematic Reviews and Meta-Analyses; Page et al., 2021) to ensure transparency. The protocol has been registered on INPLASY (INPLASY202460070).

### 2.2 Literature search

Two researchers conducted literature searches in PubMed, Web of Science, EMbase, The Cochrane Library, CNKI, Wanfang, and VIP databases, supplemented by a reference list search. The search period covered studies published from database inception to March 2024 on randomized controlled trials of brain-computer interface interventions for improving lower limb motor function in stroke patients. The English search terms were a combination of subject headings and free terms, including: Brain Computer Interfaces, Brain-Machine Interfaces, Strokes, Cerebrovascular Accident, Cerebrovascular Apoplexy, Lower Extremity, Lower Limb, Randomized Controlled Trial.

### 2.3 Inclusion and exclusion criteria

#### 2.3.1 Inclusion criteria

Included studies followed the PICOS criteria, as shown in Table 1.

**Table 1:** PICOS strategy—inclusion criteria.

PICOS	Inclusion criteria
Population	Patients clinically diagnosed with stroke and associated lower limb motor dysfunction.
Intervention	Addition of brain-computer interface intervention to conventional rehabilitation training.
Comparator	Conventional rehabilitation methods (e.g., physical therapy, manual therapy, neuromuscular electrical stimulation, psychotherapy, pharmacological treatment).
Outcomes	Lower limb motor function (FMA-LE), balance function (BBS), activities of daily living (modified Barthel index).
Study Design	Randomized controlled trial.

#### 2.3.2 Exclusion criteria

① Animal studies; ② Review articles and conference proceedings; ③ Studies involving patients with cognitive impairments, those who have received psychiatric treatment, or those with other serious diseases affecting the heart, liver, brain, or lungs were excluded; ④ Studies where data could not be extracted, and the original data could not be obtained even after contacting the authors.

### 2.4 Literature screening and data extraction

The articles retrieved from each database were imported into Endnote 20.0 software for screening and de-duplication. Two researchers independently screened the articles according to the inclusion and exclusion criteria, first reading the titles and abstracts for initial screening, then downloading the full texts of the remaining articles for further screening. After the two researchers completed the screening, they compared the articles they extracted. In case of any discrepancies or disagreements, a third researcher was involved in a discussion to decide whether to include the study.

The researchers extracted data according to a pre-established data extraction form, including: first author, country, year of publication, basic participant information, sample size, intervention details (type, duration, frequency, and cycle), and outcome measures.

### 2.5 Quality assessment

The quality of the studies was assessed using the PEDro scale (De Morton, 2009), which includes 10 criteria: ITT (intention-to-treat) analysis, random allocation, allocation concealment, baseline similarity, blinding of participants, blinding of outcome assessors, dropout rate ≤ 15%, intention-to-treat analysis, blinding of therapists, and point measurement and difference measures. One point was given for meeting each criterion, and zero points were assigned for not meeting it. The total score on the scale is 10 points: a score below 4 indicates poor quality, 4–5 points indicate moderate quality, 6–8 points indicate good quality, and 9–10 points indicate high quality.

The GRADE evidence grading system was used to assess the quality of evidence for outcome measures (Guyatt et al., 2008). The evaluation of evidence quality includes five factors that may downgrade the evidence: publication bias, inconsistency, imprecision, indirectness, and study limitations. Specifically, a downgrade of 3 levels indicates very low-quality evidence, 2 levels indicates low-quality evidence, 1 level indicates moderate-quality evidence, and no downgrade indicates high-quality evidence. The final evidence quality is categorized into four levels: high, moderate, low, and very low. The quality assessment was conducted independently by two researchers. In case of disagreement, a third researcher was involved in a discussion to reach a consensus.

### 2.6 Statistical analysis

Stata 17.0 software was used to perform effect size pooling, heterogeneity testing, subgroup analysis, and publication bias testing for all included studies’ outcome measures (Statacorp, 2021). The included outcome measures were all continuous variables. Outcome measures with the same measurement methods and units were represented by mean difference (MD) and its 95% confidence interval (CI). For outcome measures with different methods or units, standardized mean difference (SMD) and its 95% CI were used. Heterogeneity was tested using *p*-value and I^2^. If *p* < 0.05 and I^2^ > 50%, heterogeneity was considered present, and a random-effects model was used. If not, no significant heterogeneity was found between studies, and a fixed-effects model was used.

## 3 Research results

### 3.1 Literature search results

**Figure 1:**
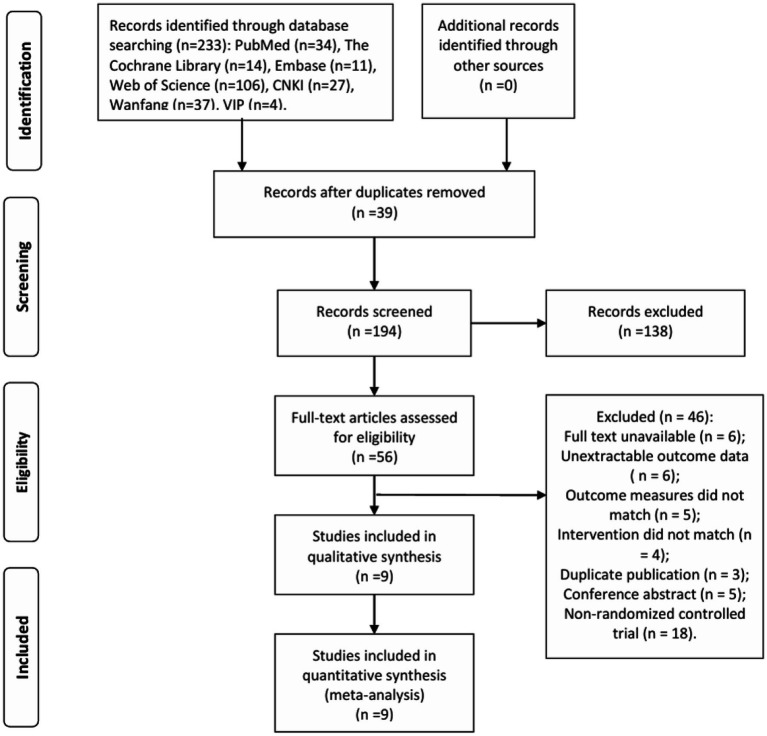
Literature screening flow.

A total of 233 relevant articles were initially retrieved, including 165 in English and 68 in Chinese. All retrieved articles were imported into Endnote 20.0, and 39 duplicate studies were removed. After reading the titles and abstracts, 138 articles were excluded, leaving 56 studies. After downloading and carefully reviewing the full texts, 46 studies were excluded, and 9 studies were ultimately included for Meta-analysis (see Figure 1).

### 3.2 Basic characteristics and intervention features of included studies

A total of 9 RCTs were included in this study, involving 419 stroke patients, with disease durations ranging from the acute phase to the post-stroke sequelae phase. The intervention in the experimental group was primarily movement imagination BCI, while the control group predominantly received conventional rehabilitation; the total intervention doses varied significantly (2.5–20 h). Notably, only one study reported complete performance metrics of the BCI system, while the others did not provide clear classification accuracy. See Table 2 for details.

**Table 2:** Characteristics of included studies.

Author	Sample size (T/C)	Age (years)	Disease course	Brain-computer interface intervention type	Comparator intervention type	Intervention dose (hours)	BCI Accuracy or classifier performance	Outcome measures
Zhao et al. (2022)	14/14	50.1 ± 11.1/ 56.1 ± 11.5	Subacute Phase	BCI Robot Training	Conventional Rehabilitation Training	12	Not Reported	FMA, MBI
Gao (2024)	32/34	53.94 ± 11.48/55.71 ± 12.54	Recovery Phase	MI-BCI Training	Conventional Rehabilitation Training	6–7	Focused Motor State Ratio (MSP): Upper Limb: 57.33 ± 28.05%; Lower Limb: 60.91 ± 26.05% Effective Training Rotation (ETR): Upper Limb: 33.22 ± 12.88 r/min; Lower Limb: 19.09 ± 6.95 r/min System Feedback Delay: Approx. 110 ms	FMA, MBI
Fang et al. (2018)	20/20	61.00 ± 5.61/61.80 ± 6.11	Recovery Phase	MI-BCI Training	Conventional Rehabilitation Training	20	“Imagery Accuracy” as the qualification standard, but specific values were not reported.	FMA, MBI
Sun (2023)	30/30	53.40 ± 13.34/54.57 ± 12.289	Recovery Phase	MI-BCI Training	Conventional Rehabilitation Training	5	Not Reported	FMA, MBI, BBS
Zhang et al. (2021)	40/40	68.37 ± 6.74/68.70 ± 6.44	Acute Phase	BCI Robot Training	Conventional Rehabilitation Training	14	Not Reported	FMA
Li and Hong (2023)	15/15	18–75	Recovery Phase	MI-BCI Training	Conventional Rehabilitation Training	2.5	Not clearly reported, only used “image and machine movement when attention is focused, stopping when not focused” as feedback mechanism	FMA
Luo (2023)	32/32	18–70	Acute Phase	MI-BCI Training	Conventional Rehabilitation Training	10	Not clearly reported, only mentioned “30% completion of motor imagery triggers therapy”	FMA, MBI
Chung et al. (2020)	13/12	52.0 ± 14.6/54.1 ± 14.7	Post-stroke Sequelae Phase	BCI-FES Training	Conventional Rehabilitation Training	7.5	“Concentration Index” used as a control signal, but the system’s classification accuracy was not reported.	BBS
Chung et al. (2015)	5/5	43.6/ 50.2	Post-stroke Sequelae Phase	BCI-FES Training	Conventional Rehabilitation Training	2.5	EEG attention index (SMR + mid-Beta/theta) used as the threshold for triggering FES. The system’s classification accuracy was not reported.	BBS

T represents the experimental group, C represents the control group, BCI stands for Brain-Computer Interface, BCI-MI refers to Brain-Computer Interface based on Motor Imagery, BCI-FES refers to Brain-Computer Interface-driven Functional Electrical Stimulation, FMA stands for Fugl-Meyer Assessment, MBI stands for Modified Barthel Index, and BBS stands for Berg Balance Scale.

### 3.3 Quality assessment of included studies

In the 9 included studies, all studies reported random allocation, intention-to-treat analysis, statistical analysis between groups, point measurements and difference measures, baseline similarity, and a dropout rate ≤ 15%. Four studies reported allocation concealment, two studies blinded participants, and two studies blinded therapists. The scores on the PEDro scale ranged from 6 to 9, with an average score of 6.9, indicating overall good study quality (see Table 3).

**Table 3:** Quality assessment of studies.

Author	Random allocation	Allocation concealment	Baseline similarity	Blinding of participants	Blinding of therapists	Blinding of outcome assessors	Participation rate > 85%	Intention-to-treat analysis	Statistical analysis between groups	Point measurement and difference measures	Total score
Zhao et al. (2022)	1	1	1	1	1	0	1	1	1	1	9
Gao (2024)	1	0	1	0	0	0	1	1	1	1	6
Fang et al. (2018)	1	0	1	0	0	0	1	1	1	1	6
Sun (2023)	1	1	1	0	1	0	1	1	1	1	8
Zhang et al. (2021)	1	0	1	0	0	0	1	1	1	1	6
Li and Hong (2023)	1	0	1	0	0	0	1	1	1	1	6
Luo (2023)	1	1	1	0	0	0	1	1	1	1	7
Chung et al. (2020)	1	1	1	1	0	0	1	1	1	1	8
Chung et al. (2015)	1	0	1	0	0	0	1	1	1	1	6

### 3.4 Meta-analysis results

#### 3.4.1 Effects of brain-computer interface training on lower limb motor function in stroke patients

**Figure 2:**
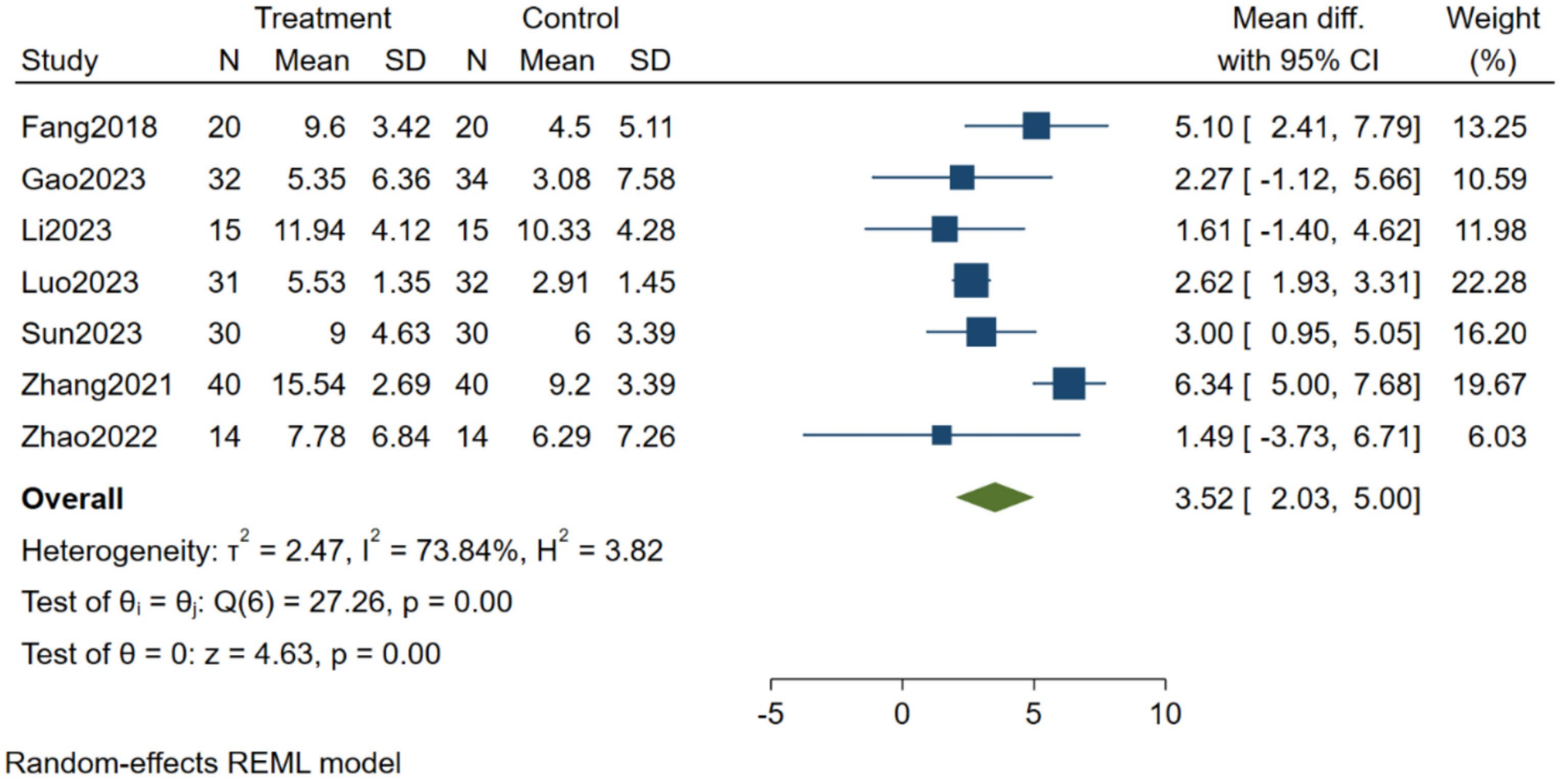
Forest plot of the effects of brain-computer interface training on lower limb motor function in stroke patients.

As shown in Figure 2, seven studies were included to compare the effects of BCI training versus control on lower limb motor function in stroke patients. Heterogeneity testing showed I^2^ = 73.84%, indicating substantial heterogeneity among the studies; therefore, a random-effects model was used for analysis. The Meta-analysis results showed MD = 3.52, 95% CI [2.03, 5.00], *p* < 0.001. These results indicate that BCI has a significant effect on lower limb motor function in stroke patients.

**Figure 3:**
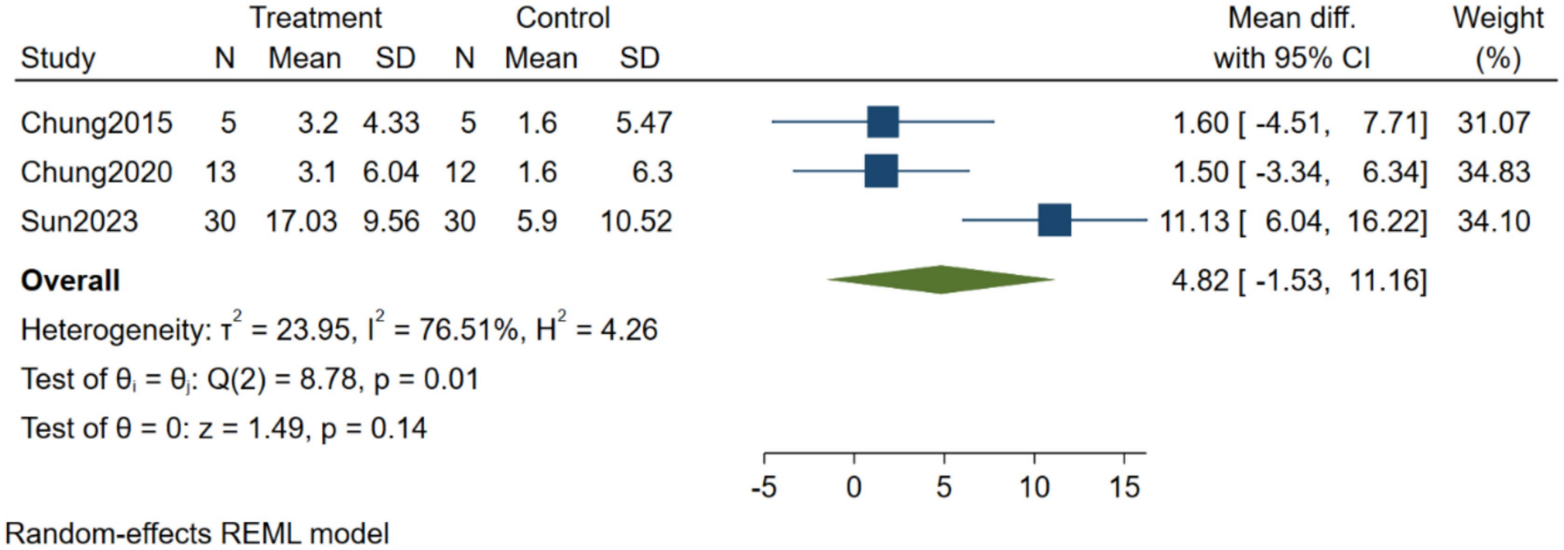
Forest plot of the effects of brain-computer interface training on balance function in stroke patients.

To explore potential sources of heterogeneity among studies, subgroup analyses were conducted based on stroke disease course, intervention type, and intervention dose. The effect sizes and heterogeneity results for each subgroup are presented in Table 4. The subgroup analysis showed that the effect size was 3.89 in the acute and subacute phases and 3.12 in the recovery phase, both of which were statistically significant. Regarding intervention type, the effect size was 2.73 for MI-BCI and 4.60 for BCI-Robot, both statistically significant. Regarding intervention dose, the effect size was 2.60 for 2.5–10 h and 5.46 for 12–20 h, both statistically significant. Stroke disease course, intervention type, and intervention dose may be sources of heterogeneity (Figure 3).

**Table 4:** Subgroup analysis results.

Study characteristic	Subgroup	Number of included studies	Effect size (95% CI)	I^2^/%
Disease course	Acute and subacute phases	3	3.89 [0.95,6.82]	90.37
	Recovery phase	4	3.12 [1.78,4.47]	2.96
Intervention type	MI-BCI	5	2.73 [2.12,3.34]	0
	BCI-Robot	2	4.60 [0.04,9.16]	67.80
Intervention dose	2.5–10 h	4	2.60 [1.97,3.23]	0
	12–20 h	3	5.46 [3.65,7.27]	33.88

#### 3.4.2 Effects of brain-computer interface training on balance function in stroke patients

**Figure 4:**
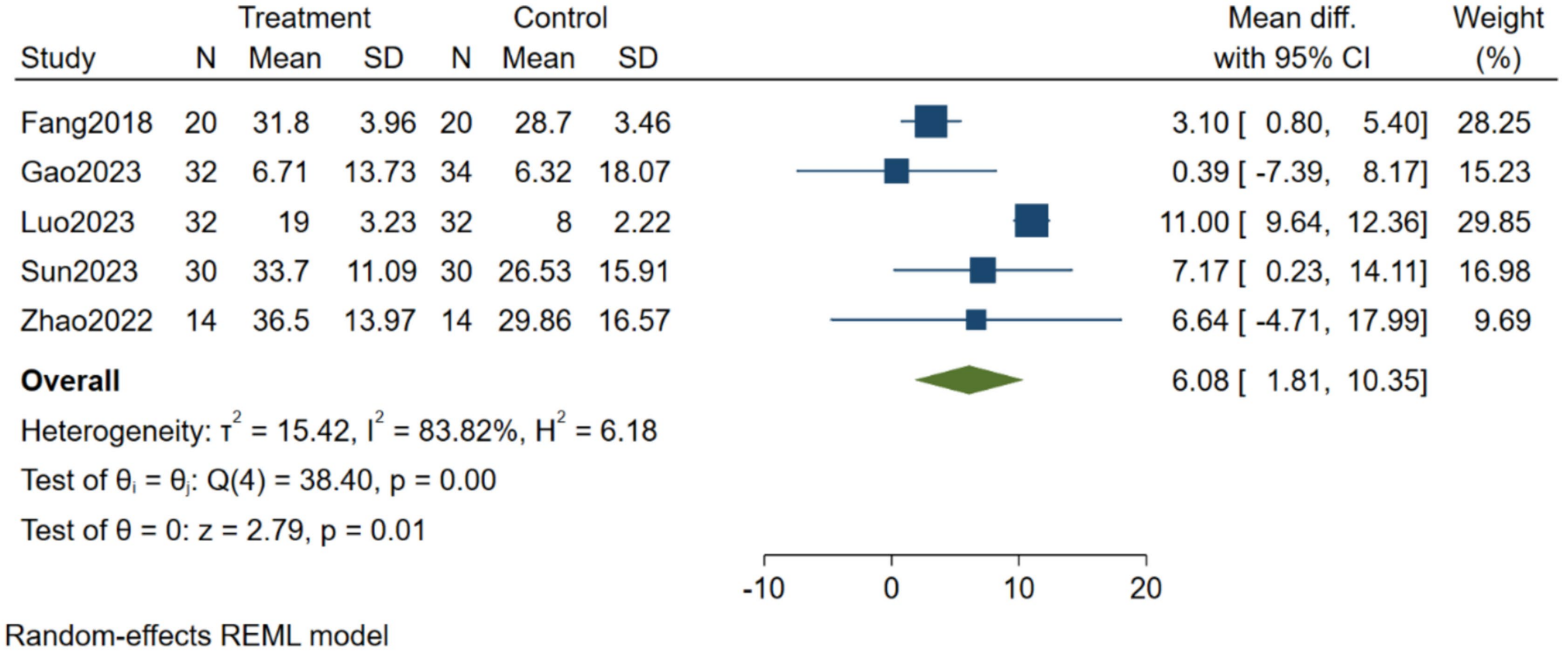
Forest plot of the effects of brain-computer interface training on activities of daily living in stroke patients.

As shown in Figure 4, three studies were included to compare the effects of BCI training versus control on balance function in stroke patients. Heterogeneity testing showed I^2^ = 76.51%, indicating substantial heterogeneity among the studies; therefore, a random-effects model was used for analysis. The Meta-analysis results showed MD = 4.82, 95% CI [−1.53, 11.16], *p* = 0.14. These results indicate that BCI has no significant effect on balance function in stroke patients.

#### 3.4.3 Effects of brain-computer interface training on activities of daily living in stroke patients

**Figure 5:**
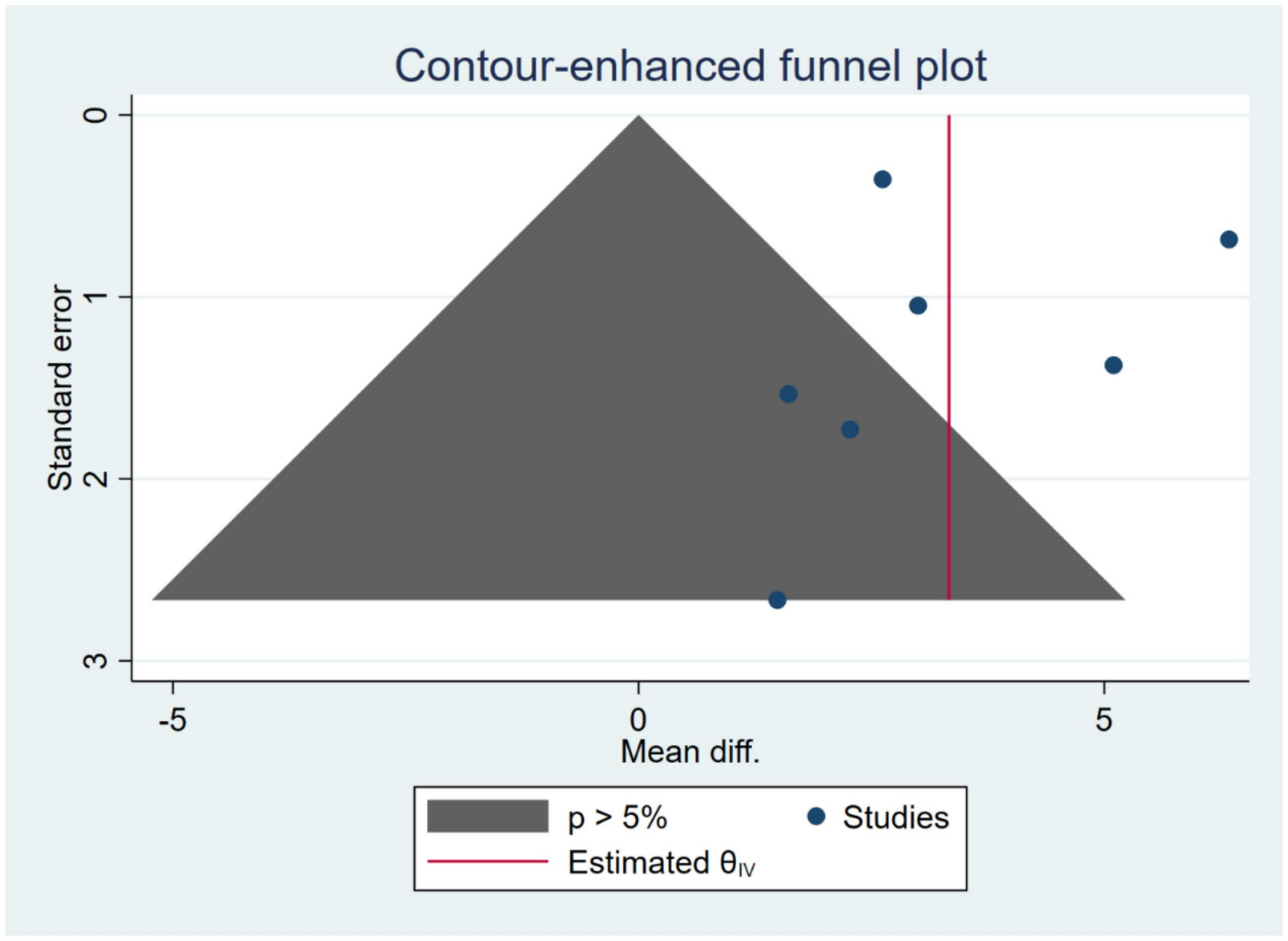
Publication bias for lower limb motor function.

As shown in Figure 5, five studies were included to compare the effects of BCI training versus control on activities of daily living in stroke patients. Heterogeneity testing showed I^2^ = 83.82%, indicating substantial heterogeneity among the studies; therefore, a random-effects model was used for analysis. The Meta-analysis results showed MD = 6.08, 95% CI [1.81, 10.35], *p* = 0.01. These results indicate that BCI has a significant effect on activities of daily living in stroke patients.

### 3.5 Publication bias

In this study, publication bias was analyzed for the lower limb motor function outcome. The results showed that the scatter points representing individual studies were roughly symmetrically distributed on both sides of the pooled effect size. More importantly, most of the scatter points were evenly distributed in the non-significant region (*p* > 0.05), with no large-scale absence of research points in this region. This suggests that although there is substantial variability in the results of small sample studies, there is no clear evidence of systematic omission of negative results (i.e., studies that are statistically non-significant). The Egger test results showed a beta1 coefficient of −1.06 (SE = 1.283), a test statistic z = −0.82, and *p* = 0.4096, indicating that there is no significant publication bias in the current Meta-analysis.

### 3.6 Evidence quality assessment

**Figure 6:**
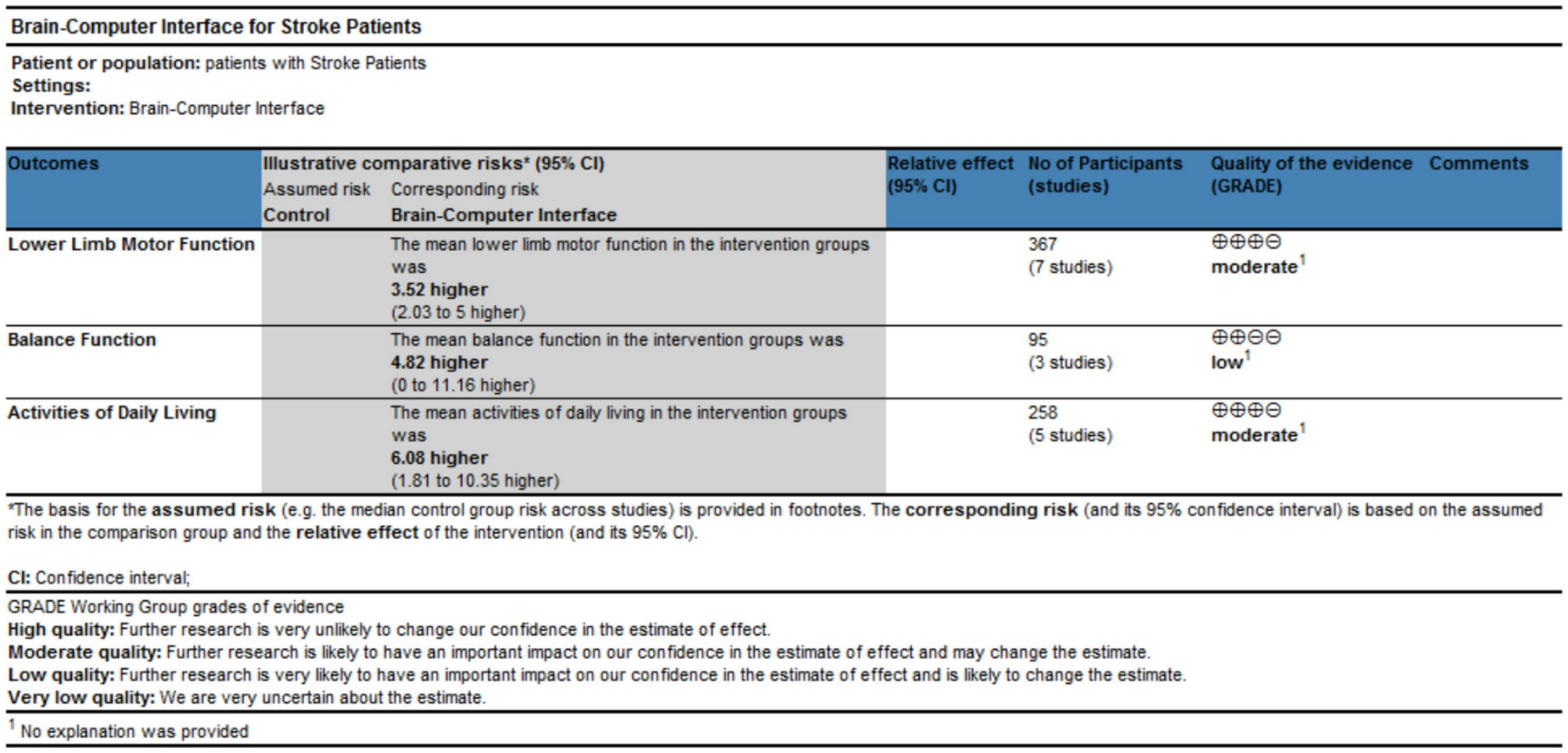
Evidence quality assessment.

The GRADEPro software (Figure 6) indicates that the evidence quality for lower limb motor function and activities of daily living is moderate, while that for balance function is low. There is high heterogeneity for all outcomes, and the lack of allocation concealment and incomplete blinding in most studies were the main reasons for downgrading.

## 4 Discussion

This study found that Brain-Computer Interface (BCI) training can improve lower limb motor function and activities of daily living in stroke patients. The potential neural mechanisms underlying BCI’s effect on motor function recovery in stroke patients may involve the induction of physiological and structural neural plasticity, specifically the reorganization of the damaged cerebral cortex. These functional and structural reorganizations are reflected in the modulation of spontaneous brain activity, the induction of brain activity excitability (Soekadar et al., 2011; Van Dokkum et al., 2015), the imbalance in microstructural hemispheric connectivity (Ang et al., 2015; Song et al., 2014; Ramos-Murguialday et al., 2013), and the cortical–subcortical connectivity in motor-related brain regions (Liew et al., 2016). In most studies, such changes have been shown to improve motor function in stroke patients. This study also found that BCI training had no significant effect on improving balance function in stroke patients. A possible reason is the limited number of studies included and the substantial heterogeneity, which may have impacted the stability of the results. Future studies should include more high-quality, large-sample randomized controlled trials to verify these findings.

To interpret the clinical implications of these findings, we referred to the minimal clinically important difference (MCID) thresholds for various functional dimensions. However, as summarized by Nakazono et al., a robust and widely accepted MCID threshold for the Fugl-Meyer Assessment of the Lower Extremity (FMA-LE) in patients with acute and subacute stroke remains elusive (Nakazono et al., 2025). Consequently, although this meta-analysis confirms a positive effect of BCI on lower limb motor function, the clinical relevance of the effect size in the acute-subacute population remains difficult to quantify. Notably, several controlled studies on MI-BCI for lower-limb rehabilitation have demonstrated functional improvements that meet or exceed established MCID thresholds when the intervention ensures adequate contingency, appropriate timing, and sufficient training dose (Mrachacz-Kersting et al., 2016; Chung et al., 2015; Ang et al., 2015; Sebastián-Romagosa et al., 2023), suggesting its potential clinical value. In terms of activities of daily living, referencing the MCID of 1.85 points for the Barthel Index established by Hsieh et al. (2007), the effect size in this study was 6.08, significantly surpassing this standard, indicating that BCI training likely has clear clinical value in enhancing patients’ real-life functional abilities. Possible reasons for this difference include: heterogeneity in the patient population, variations in BCI system performance, and insufficient total intervention dose and duration in some studies, which may have limited the full translation of neuroplasticity into significant improvements in motor function. Future research should focus on optimizing BCI protocols to achieve clinically meaningful effects across all functional dimensions.

This study found that BCI was effective in improving lower limb motor function in stroke patients across different stages of stroke (acute, subacute, and recovery). Both BCI-MI and BCI-robot interventions were effective, and intervention doses of 2.5–10 h and 12–20 h were both effective. Compared to traditional rehabilitation methods, EEG-based BCI can analyze brain electrical signals through external output devices and convert them into corresponding commands, thereby facilitating interaction between the brain and the external environment (Daly and Huggins, 2015). The neurophysiological mechanism of stroke rehabilitation is the plasticity of the central nervous system. Repetitive feedback stimulation during learning and training can strengthen the connections between neuronal synapses, leading to progressive repair, compensation, and reconstruction effects in the cortical regions of the brain (Carvalho et al., 2019). Several studies have shown that in acute or subacute stroke patients, robotic devices combined with physical therapy in enhanced training protocols produce considerable gains in motor function (Moucheboeuf et al., 2020; Kakuda, 2020). However, end effectors and exoskeleton devices provide only passive training, and patients cannot perform voluntary movements (Louie and Eng, 2016). A BCI-controlled lower limb rehabilitation system enables stroke patients to actively train their lower limbs, thus improving lower limb motor function. This study also found that the effect size for interventions in the acute and subacute stages was higher than that in the recovery stage. The effect size for BCI-Robot was higher than that for MI-BCI, and the effect size for 12–20 h of intervention was higher than that for 2.5–10 h, with the effect size for 12–20 h reaching 5.46, which is close to the FMA-LE MCID threshold. Current evidence suggests that BCI therapy should be prioritized in the early phase of stroke to leverage the critical window of neuroplasticity, with a recommended intervention dose exceeding 12 h. Nevertheless, it is important to note that although the BCI-Robot subgroup yielded a larger pooled effect size (MD = 4.60), this estimate is unstable, being derived from only two studies and exhibiting substantial heterogeneity (I^2^ = 67.8%). This suggests that the treatment effect is highly variable and may depend on specific device characteristics, study populations, and intervention protocols. In contrast, BCI-MI demonstrates a smaller but more consistent effect (MD = 2.73, I^2^ = 0%), indicating higher reproducibility across trials. Therefore, future randomized controlled trials with standardized stimulation protocols and larger sample sizes are required to confirm whether BCI-Robot provides a genuinely superior clinical benefit.

In addition to efficacy outcomes, the feasibility of implementing BCI-based interventions in clinical or community settings is also worth exploring. The preparation time required for BCI systems remains a practical barrier to clinical efficiency. However, the included studies reported high patient participation rates, low dropout rates, and high compliance, with no adverse events reported, indicating the safety and acceptability of this method. The heterogeneity in total intervention dosage (ranging from 2.5 to 20 h) suggests that the intervention is flexible and can be adapted to different rehabilitation stages. Shorter duration, higher frequency protocols may be suitable for hospitalized acute-phase patients, while longer-term training may benefit chronic-phase patients in the community. Future implementation studies should focus on simplifying operational procedures, developing optimal dosage guidelines, and conducting formal cost-effectiveness analyses to comprehensively evaluate the practical value of BCI-mediated stroke rehabilitation.

The systematic review included nine studies. The methodological quality, as assessed by the predefined scale, yielded an average score of 6.9, which corresponds to a moderate-to-low certainty of evidence. However, due to the specific nature of BCI training, there are inherent difficulties in implementing allocation concealment and blinding of both researchers and therapists, which may affect the reliability of some study results. In terms of heterogeneity, the results of the meta-analysis for lower limb motor function, balance function, and activities of daily living showed I^2^ statistics greater than 50%, indicating high heterogeneity among studies. Subgroup analysis of lower limb motor function revealed that the stroke patient’s disease stage, BCI intervention type, and total intervention dose might be potential sources of heterogeneity. Publication bias analysis of the lower limb motor function outcome showed no significant publication bias. Due to the insufficient number of studies included, subgroup analysis and publication bias testing for balance function and activities of daily living were not conducted. The GRADE system was used to assess the quality of evidence, and no significant downgrading factors were found in terms of indirectness and imprecision. Ultimately, the quality of evidence for lower limb motor function and activities of daily living was rated as moderate, while the quality of evidence for balance function was rated as low. This was primarily limited by the aforementioned risk of bias and heterogeneity.

This study also has several limitations: (1) the interventions in this study included BCI-MI, BCI-FES, and BCI-robot, and the effectiveness of BCI in this study may not be generalized to all types of BCI. (2) The methodological reporting quality limits the reliability of the results. Only one study reported the online classification accuracy or performance of the BCI system, while other studies did not provide complete reports. This metric is crucial for ensuring that the treatment dose aligns with the intended outcome, and its absence is a core limitation. (3) The geographic distribution of the studies was highly concentrated in Asia (China, Korea), and their rehabilitation systems and cultural contexts may differ from those in Western countries, which to some extent impacts the external validity and generalizability of the study conclusions. (4) This study primarily employed the FMA-LE as the main outcome, with balance function and activities of daily living as secondary outcomes, and did not include an in-depth analysis of functional gait metrics. Given that the FMA-LE may have limited ecological validity for assessing lower-limb function, future trials and meta-analyses should incorporate functional gait outcomes. The absence of these measures in the current evidence base restricts a comprehensive clinical interpretation of the findings. (5) Most studies did not report follow-up data, and the durability of BCI-induced benefits remains largely unknown. Future research should include more high-quality, large-sample, long-term follow-up randomized controlled trials to further validate these findings.

## 5 Conclusion

Current evidence suggests that BCI-based interventions have a beneficial effect on lower limb motor function and activities of daily living in stroke patients. Interventions initiated during the acute or subacute phase, with a total dose exceeding 12 h, appear to be associated with superior outcomes. However, the certainty of this evidence is moderate to low, necessitating further validation. Future research should prioritize large-scale, high-quality randomized controlled trials to definitively establish the efficacy of BCI technology and elucidate its optimal implementation protocols.
